# Association Between Vitamin D Level and Neonatal Respiratory Distress Syndrome: A Systematic Review and Meta-Analysis

**DOI:** 10.3389/fped.2021.803143

**Published:** 2022-01-21

**Authors:** Rina Zang, Yayu Zhang, Hanshuo Zhang, Xueyi Zhang, Yuening Lv, Dan Li

**Affiliations:** Department of Neonatology, Affiliated Hospital of Inner Mongolia Medical University, Huhhot, China

**Keywords:** neonatal respiratory distress syndrome, vitamin D, meta-analysis, NRDS, systematic review

## Abstract

**Background:**

In recent years, vitamin D in the occurrence of lung diseases has gradually become a hot topic. Although the role of vitamin D in normal lung development has been confirmed, the correlation between vitamin D level and neonatal respiratory distress syndrome (NRDS) is not clear.

**Objective:**

To evaluate the association between vitamin D level and NRDS.

**Methods:**

We performed a comprehensive search of the following electronic databases: PubMed, Embase, Cochrane Library, Web of Science, and China National Knowledge Infrastructure (CNKI). Literature screening and quality assessment were performed according to inclusion and exclusion criteria. The Newcastle–Ottawa Scale was used to assess the methodological components of each study, and Stata 15.1 software to perform the Meta-analysis.

**Results:**

A total of nine case-control studies were included, with 653 infants with NRDS and 501 infants without NRDS. The Meta-analysis showed no heterogeneity across all studies(I^2^=0.0%, P=0.583). The fixed-effect model showed that 25 hydroxy vitamin D level of children in the NRDS group was significantly lower than that of the non-NRDS group(SMD = −0.51, 95%CI: −0.63 to −0.39, *p* ≤ 0.05).

**Conclusion:**

This systematic review and meta-analysis study suggests that vitamin D deficiency is very likely to be a high-risk factor of NRDS, and reasonable vitamin D supplementation during pregnancy and after birth is of great significance.

## Introduction

Neonatal respiratory distress syndrome (NRDS), formerly known as hyaline membrane disease (HMD), is a clinical syndrome characterized by respiratory distress and progressive aggravation soon after birth (1), mostly due to insufficient pulmonary surfactant secreted by alveolar type II epithelial cells, which is more common in premature infants in clinical practice (2). Neonates with younger gestational age tend to suffer from a higher incidence rate, lighter weight, and a higher fatality rate (3). Vitamin D is a fat-soluble steroid derivative that plays very important physiological functions in the human body (4), namely improving the body's absorption of calcium and phosphorus, promoting growth and bone calcification (5), and regulating the body's immune function (6). With more and more in-depth research at home and abroad in recent years, many animal and laboratory studies have found that the lack of vitamin D is also involved in the occurrence of respiratory diseases in children (7–9). NRDS is one of the most common severe lung diseases in newborns. Recently, some scholars have speculated that the occurrence of NRDS may be related to vitamin D deficiency, but this hypothesis has not been confirmed and elucidated. We assumed that vitamin D deficiency is associated with neonatal respiratory distress syndrome, for which we perform this meta-analysis to provide evidence-based medicine for the prevention and treatment of NRDS.

## Methods

This analysis was performed on the Preferred Reporting Items for Systematic Review and Meta-Analyses (PRISMA) statement (10). All analyses involved were based on previously published studies, and no ethical approval and patient consent were required.

### Search Strategy and Selection Criteria

We searched PubMed, Embase, Cochrane Library, Web of Science, and China National Knowledge Infrastructure (CNKI) databases from inception to August 23, 2021. The search terms were as follows: “vitamin D” or “25-hydroxyvitamin D” or “25-hydroxyergocalciferol” or “25(OH)D” or “1,25(OH)2-vitD” or “ergocalciferol” or “cholecalciferol” or “hydroxycholecalciferol” or “calcifediol” or “dihydroxycholecalciferol”; and “Neonatal respiratory distress syndrome ” or “NRDS ” or “Neonatal hyaline membrane disease” or “NHMD”. We used the combination of subject words and free words to perform the search process, and logical symbols, wildcards, Boolean logic operators to write search expressions. The detailed search strategy for PubMed was shown in Table 1. Reference lists of the relevant articles were additionally reviewed for any further relevant studies. The search was notrestricted by language.

**Table 1:** Search strategy for PubMed.

**NO**.	**Search query**	**Results**
#1	Search: **“Respiratory Distress Syndrome, Newborn”[Mesh]** Sort by: **Most Recent**	15,452
#2	Search: **[(Infantile Respiratory Distress Syndrome) OR (Neonatal Respiratory Distress Syndrome)] OR (Respiratory Distress Syndrome, Infant)**	20,698
#3	Search: **neonatal hyaline membrane disease**	3,101
#4	Search: **NRDS**	148
**#**5	Search: **NHMD**	16
#6	**#1 OR #2 OR #3 OR #4 OR #5**	**21,129**
#7	Search: **“Vitamin D”[Mesh]** Sort by: **Most Recent**	62,860
#8	Search: **25-hydroxyvitamin D[Supplementary Concept]**	8,061
**#9**	Search: **((((((((25-hydroxyvitamin D) OR (25-hydroxyergocalciferol)) OR (25(OH)D)) OR (1,25(OH)2-vitD)) OR (ergocalciferol)) OR (cholecalciferol)) OR (hydroxycholecalciferol)) OR (calcifediol)) OR (dihydroxycholecalciferol)**	43,786
#10	**#7 OR #8 OR #9**	**66,818**
#11	**#6 AND #11**	**32**

*Bold values represent the final results of searches by logical sentences*.

### Study Selection

The studies included for this review were: (a) studies that were all case-control studies;(b) subjects were premature infants (gestational age <37 weeks), and all had venous blood drawn within 24 h after birth; (c) all case group children met the diagnostic criteria of NRDS in the 2016 edition of the European consensus guidelines for the management of NRDS (11) [except one (12)]; (d) data reported in the literature are independent research results. If more than two studies with similar research objects and data were retrieved at the same time, the latest published literature prevailed. Exclusion criteria were as follows: (a) the quality of the literature was poor, including serious flaws in research design, unclear outcome indicators, etc. (b) the research types were case reports, case series, animal experiments, *in vitro* experiments, meeting minutes, reviews. (c) literature did not have access to the original text, raw data, or any further relevant information.

### Data Extraction and Outcomes

Two authors (Zang RN and Zhang YY) independently screened the searched literature according to inclusion and exclusion criteria, then extracted data and checked with each other. The controversial literature was evaluated by a third author for inclusion. When possible, the author of the literature was contacted in the case of lack of information. When selecting documents, the titles and abstracts would be first read to exclude irrelevant documents, and then the full text would be read to determine whether they could be included. The main content of data extraction was: (1) basic information of the research, including the first author, publication time, mean gestational age and birth weight, etc.; (2) basic characteristics and important outcome indicators of the research objects, including study time, study location, sample size, 25 hydroxy vitamin D (25(OH)D) level, etc.

We also used the Newcastle-Ottawa Scale (NOS) to evaluate the methodological quality and risk of bias of included literature. The scale includes three columns including the selection of study subjects, comparability between groups, exposure or outcome evaluation, with a maximum score of nine points. A high-quality study is counted as greater than six points. Two authors (Zang RN and Zhang YY) worked independently and any disagreements were resolved by discussion and consensus.

### Statistical Analysis

This meta-analysis was performed by Stata 15.1 (Stata Corporation). Data were used as input and double-checked by two reviewers. Data syntheses and interpretations were conjointly performed by two authors to make sure the accuracy of the results. 25(OH)D level was the measurement data, so standard mean difference (SMD) and its 95% confidence interval (CI) were used as the effect quantity. Chi-Squared test was used to analyze the heterogeneity of the included studies, and *I*^2^ to judge it.

If the *P* ≥ 0.1 and the *I*^2^ ≤ 50%, it is suggested that there was no statistical heterogeneity or the heterogeneity is small among the studies. Conversely using the fixed-effect model to analyze if the *P* < 0.1 and the *I*^2^ > 50%, it is suggested that there was a statistical heterogeneity among the studies, and using the random-effect model to analyze. There was a statistical significance if the *P* < 0.05. For obvious clinical heterogeneity, subgroup analysis or sensitivity analysis was used. Publication bias was evaluated by using funnel plots, and asymmetry was assessed by conducting an Egger regression test. For funnel plot asymmetry, *P* < 0.05 was considered significantly different.

## Results

### Study Selection

**Figure 1:**
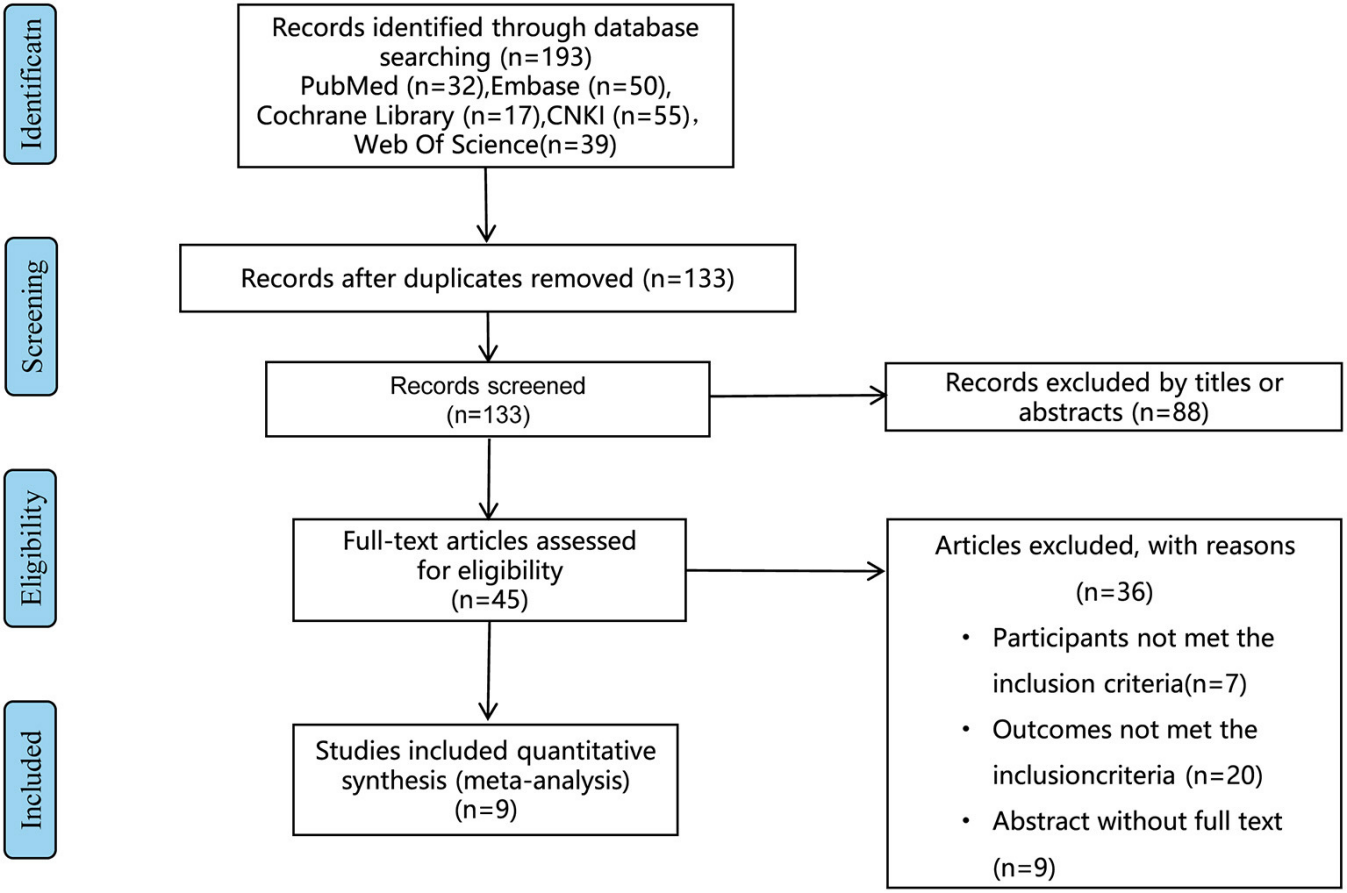
The PRISMA flow diagram for the study selection process.

Studies concerning the vitamin D level and NRDS were considered for inclusion. The exclusion criteria were shown in the flow chart (Figure 1). After careful and discreet evaluation based on the included and excluded criteria, nine studies were included (12–20).

### Study Characteristics

A total of 1,154 premature babies were included in this meta-analysis, of which 653 were diagnosed with NRDS and 501 were not. The included studies were all case-control studies and there was no difference in the diagnostic criteria of NRDS among these studies. There was no statistically significant difference in gender, gestational age, and birth weight composition between the NRDS group and the non-NRDS group. The basic characteristics of the included literature are shown in Table 2.

**Table 2:** Characteristics of studies included in this meta-analysis.

				**Case group/control group (NRDS /Non-ARDS)**					
**References**	**Country**	**Period**	**Design**	**Number of Patients**	**Sex (male/ female)**	**Gestational age (week)**	**Birth weight (gr)**	**Vitamin D levels (ng/ml)**	**NOS score**
Yu et al. (13)	China	2014.01– 2016.12	Case-control study	72/40	(24/16)/ (52/20)	29.9 ± 0.9/ 29.3 ± 2.1	1444 ± 178/ 1392 ± 403	36 ± 11/ 45 ± 17	8
Geng et al. (14)	China	2018.09– 2019.03	Case-control study	131/29	—	33.5 ± 1.9/ 35.1 ± 1.3	2070 ± 480/ 2040 ± 540	66.37 ± 51.95/ 93.69 ± 36.98	7
Zeng et al. (15)	China	2017.03– 2019.01	Case-control study	156/40	(96/60)/ (23/17)	33.88 ± 1.71/ 34.81 ± 2.02	2210 ± 460/ 2530 ± 520	64.27 ± 36.78/ 87.59 ± 44.21	8
Al-Beltagi et al. (16)	Egypt	2016.01– 2018.12	Case-control study	96/100	(58/38)/ (61/39)	31.57 ± 1.49/ 32 ± 1.63	1862.6 ± 347.71/ 2109.8 ± 391.75	29.18 ± 21.62/ 44.68 ± 31.32	8
Boskabadi et al. (17)	Iran	2015– 2016	Case-control study	80/80	(35/45)/ (48/32)	31.57 ± 1.49/ 32 ± 1.63	1362.6 ± 347.71/ 1589.8 ± 391.75	29.18 ± 21.62/ 44.68 ± 31.32	7
Dogan et al. (18)	Turkey	2014.01– 2015.01	Case-control study	49/23	(23/26)/ (13/10)	9 ± 1.75/ 30 ± 1.5	1020 ± 410/ 1400 ± 596.25	18.72 ± 12.23/ 23.96 ± 14.23	7
Ataseven et al. (12)	Turkey	2012.10– 2013.06	Case-control study	35/117	(17/18)/ (54/63)	32 ± 1.5/ 33 ± 1.5	1667 ± 505/ 1974 ± 585	18.72 ± 12.23/ 23.96 ± 14.23	8
Yang et al. (19)	China	2015.01– 2016.01	Case-control study	34/72	—	—	—	36.43 ± 17.27/ 39.36 ± 19.06	8
Zhang et al. (20)	China	2018.03– 2020.05	Case-control study	136/43	(77/56)/ (26/17)	28.7 ± 1.78/ 31.7 ± 2.15	1 438 ± 261/ 1 590 ± 338	28.20 ± 17.97/ 39.64 ± 20.97	8

### Literature Quality Evaluation

The NOS was used to evaluate the quality of the literature. The scores of the included nine studies are >6 points, all of which were high-quality literature. Their specific scores of quality were presented in Table 3.

**Table 3:** NOS score of studies included in this meta-analysis.

	**Selection**				**Comparability**		**Exposure**			**Total**
**References**	**1**	**2**	**3**	**4**			**1**	**2**	**3**	
Yu et al. (13)	★	★	✩	★	★	★	★	★	★	8
Geng et al. (14)	★	★	✩	★	★	✩	★	★	★	7
Zeng et al. (15)	★	★	✩	★	★	★	★	★	★	8
Al-Beltagi et al. (16)	★	★	✩	★	★	★	★	★	★	8
Boskabadi et al. (17)	★	★	✩	★	★	✩	★	★	★	7
Dogan et al. (18)	★	★	✩	★	★	✩	★	★	★	7
Ataseven et al. (12)	★	★	✩	★	★	★	★	★	★	8
Yang et al. (19)	★	★	✩	★	★	★	★	★	★	8
Zhang et al. (20)	★	★	✩	★	★	★	★	★	★	8

### Assessment of Outcomes

**Figure 2:**
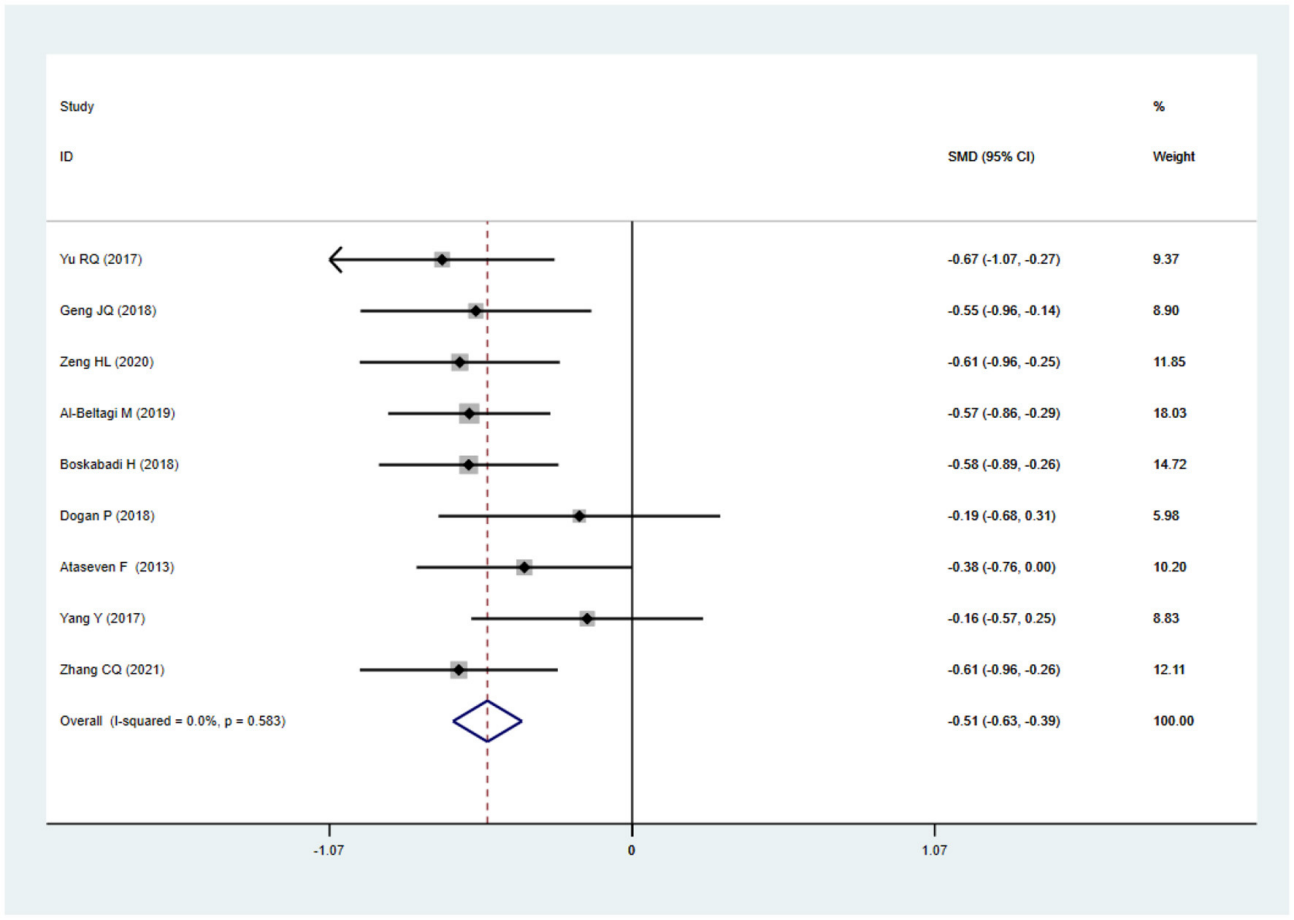
Meta analysis of the difference of 25(OH)D level between the two groups.

**Figure 3:**
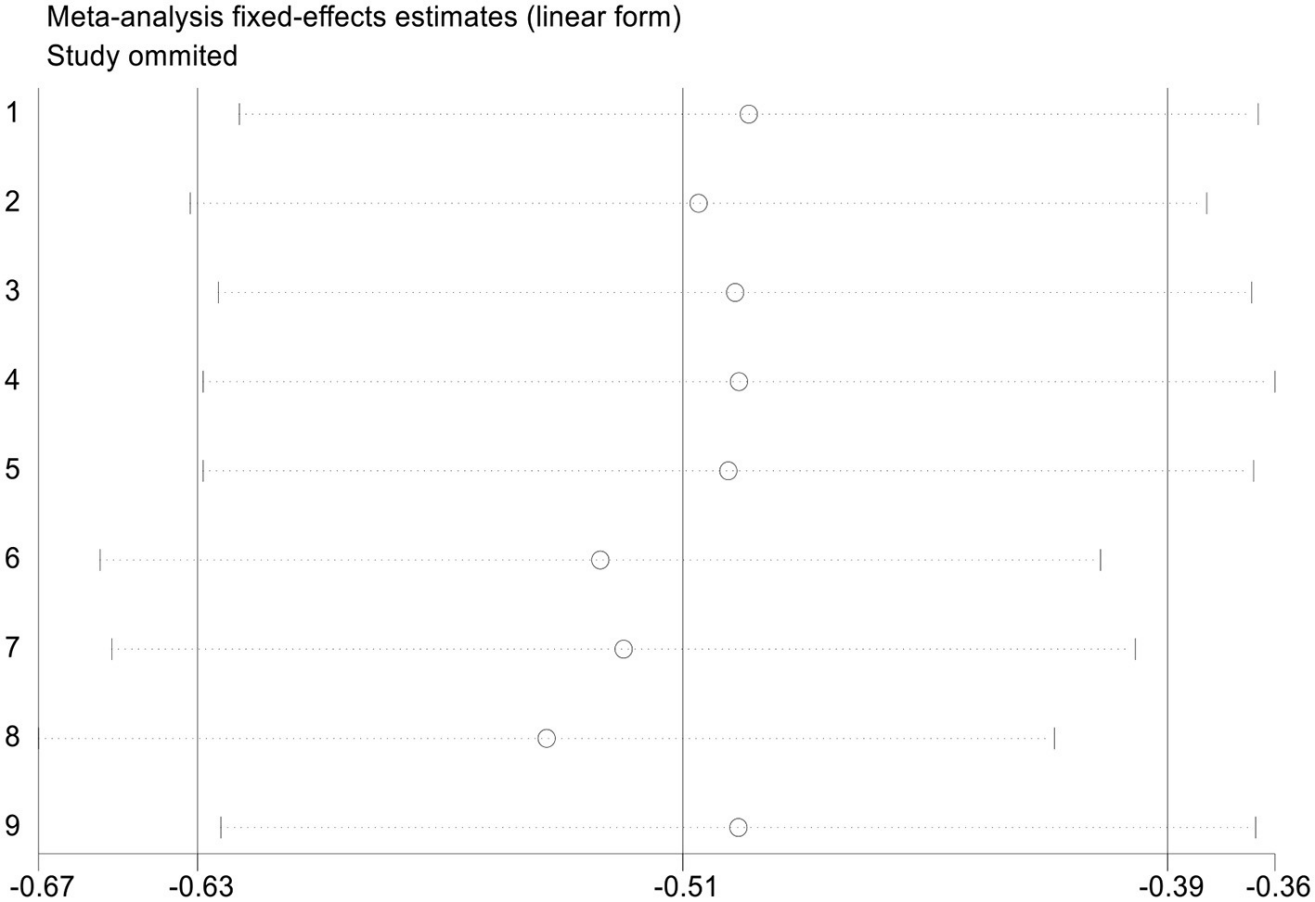
Sensitivity analysis of Association between vitamin D level and NRDS.

**Figure 4:**
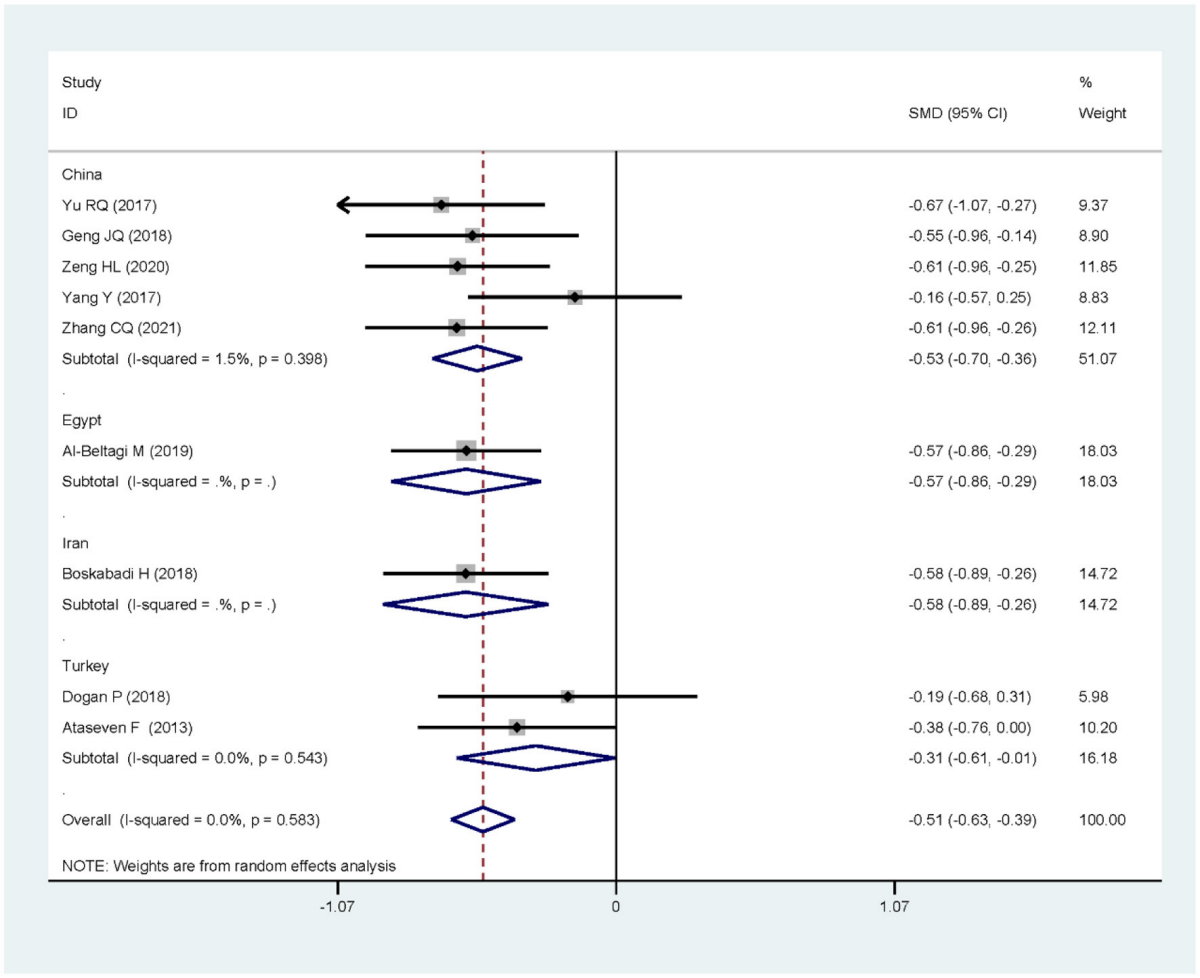
Result of subgroup analysis categorized in countries.

The nine articles in this study were tested for heterogeneity, *I* = 0.0% < 50%, and the Q test showed that *P* = 0.583 > 0.1, suggesting that there was no heterogeneity among these articles, so the fixed-effect model was used for analysis and showed that the SMD value was −0.51, the 95% confidence zone was −0.63 to −0.39 and *p* = 0.000 < 0.05, and there was statistically significant differences between the two groups, suggesting that 25(OH)D level of children in the NRDS group was significantly lower than that of the non-NRDS group (Figure 2). To ensure the accuracy and stability of the research, we further conducted a sensitivity analysis. The results showed that none of the articles caused great interference to the results of this meta-analysis, which means that the research has good stability (Figure 3). To understand the differences of VD in NRDS and non-NRDS infants between China and other countries, all data were categorized in countries to perform a subgroup analysis (Figure 4). The results of sub-group analysis show that in China, Egypt, and Iran, the 25(OH)D level of children in the NRDS group was significantly lower than the non-NRDS group, while the data of Turkey showed that there is no statistically significant difference between experimental group and controls.

### Bias Test

**Figure 5:**
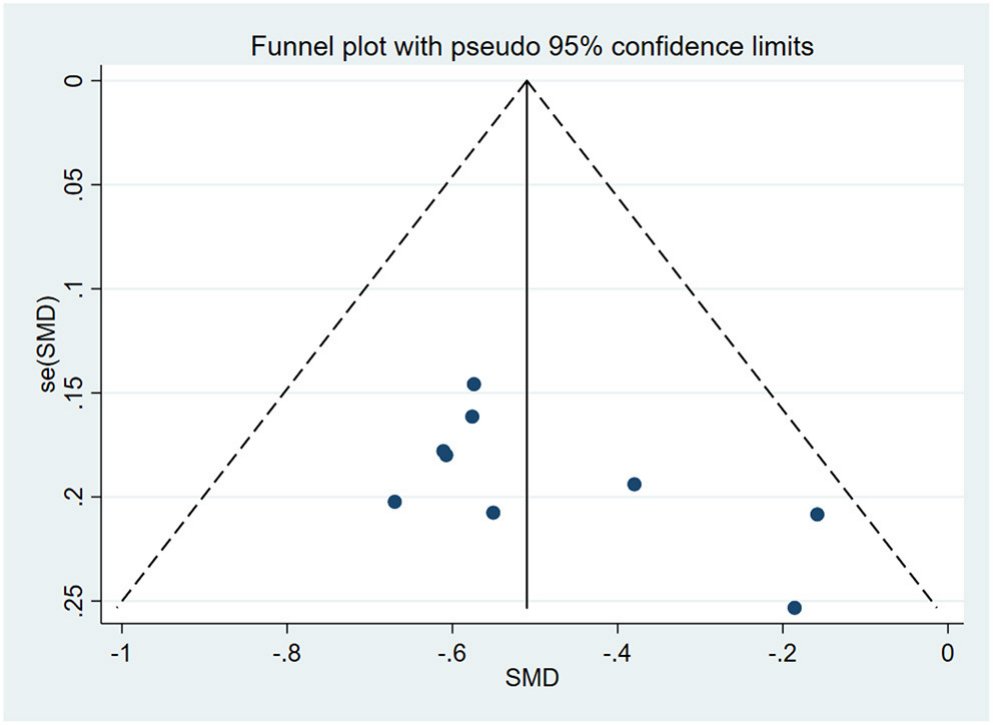
Funnel plot of Association between vitamin D level and NRDS.

The funnel plot was used to evaluate publication bias. As shown in Figure 5, it can be seen that it was symmetrical. Furthermore, the Egger regression test was performed to assess asymmetry and it was concluded that *P* = 0.087 > 0.05. Therefore, it was believed that there was no publication bias in the articles of this study.

## Discussion

The effect of vitamin D on lung development and maturation and early lung diseases in life is an emerging research field. Some animal and laboratory data indicate that vitamin D deficiency is a risk factor for NRDS but in fact, there are still few clinical studies in this area. Yu et al. (13) have found that low serum vitamin D at birth is an independent risk factor for NRDS. In addition, Dogan et al. (18) have confirmed that higher serum vitamin D levels in preterm infants can prevent NRDS. However, in contrast to this, in the latest prospective cohort study, when the cut-off value of 10 ng/ml was used, it was not found that the vitamin D status of preterm infants was related to NRDS, that is, the serum vitamin D level of preterm infants neither increased nor decreased the incidence of NRDS, which was contrary to the results of the above studies (21). Therefore, their relevance is still very controversial. In this paper, meta-analysis was used to explore the correlation between vitamin D level and NRDS risk. A total of nine high-quality articles were included. The results of the meta-analysis showed that 25(OH)D level of children in the NRDS group was significantly lower than that of the non-NRDS group, suggesting that vitamin D deficiency is very likely to be a high-risk factor for NRDS.

The specific mechanism of vitamin D's involvement in the occurrence of NRDS is still unclear. Summarizing the domestic and foreign data in recent years, it may include the following aspects: (1)Vitamin D is a steroid hormone, which can regulate the development of the fetal lung and induce the synthesis of phosphatidylglycerol and phosphatidylcholine by binding with the corresponding receptors of type II alveolar epithelial cells to promote the synthesis and secretion of surfactant in alveolar epithelial cells. Insufficient or lack of vitamin D will affect the maturation of fetal lungs to a certain extent. (2)Vitamin D deficiency can lead to down-regulation of the expression of airway proliferation proteins, leading to the inflammatory reaction, small airway spasm, reduction of alveolar surface tension, and alveolar collapse. (3)Vitamin D deficiency can induce oxidative stress and stimulate the body to produce a large amount of oxygen free radicals, which in turn cause lipid peroxidation damage in the respiratory tract and alveoli. (4) Others: some studies believe that the occurrence of NRDS is related to vitamin D's inhibition of the mRNA and protein expression of the PDGF-A gene in lung tissue, and low-level gene expression will inhibit lung development (22). Other scholars believe that the occurrence of NRDS is related to the polymorphism of vitamin D receptor genes (ApaI, BsmI, FokI, and TaqI) (23).

At present, there are few studies on vitamin D treatment of NRDS patients. Al-Beltagi et al. (16)divided 96 children with NRDS into three subgroups: conventional treatment (without vitamin D supplementation), conventional treatment combined with vitamin D supplementation of 400 IU/d, and conventional treatment combined with vitamin D supplementation of 800 IU/d. It was found that there was no significant difference in Downes respiratory distress score and PaCO_2_ among the three groups before treatment, but on the 6th day of treatment, the Downes respiratory distress score and PaCO_2_ of children with additional vitamin D supplementation in the two groups were significantly lower than those in the conventional treatment group, the hospital stay was shorter, and the incidence of complications was lower. The difference was more significant in the vitamin D supplementation group of 800 IU/d than in the 400 IU/d group. Therefore, 800 IU/d vitamin D is recommended for children with NRDS. The results of Hou et al. (24) proved that additional supplementation of 1000IU/d vitamin D can significantly reduce the disease severity of preterm infants with NRDS, shorten their nCPAP use time, mechanical ventilation time, total oxygen use time, and hospital stay. Regarding the supplemental dosage of vitamin D in newborns, there is no unified guideline at this time. American IOM guidelines (25) suggest that children should start to supplement vitamin D of 400 IU/d a few days after birth and continue to adolescence. The European Endocrine Association guidelines (3) recommend that infants supplement at least 400 IU of vitamin D every day for 6 weeks, which can improve the nutritional status of vitamin D without excessive vitamin D. The consensus of Chinese experts (26) suggests that newborns should supplement vitamin D of 400 ~ 800 IU/d as soon as possible after birth. Preterm infants, low birth weight infants, and multiple fetuses should take oral vitamin D preparation of 800IU/d from 1 week after birth and 400IU/d after three months and if premature infant formula is used, vitamin D can be reduced to 400 IU/d. The maintenance time is not uniform, mostly for 2 years.

## Limitations

Although the results of this study are consistent with previous studies, there are still some limitations: (1) The nine articles included are from different countries, so there are regional and ethnic differences. (2) There are certain differences in the gestational age, birth weight, and specific time of blood draw of the included research subjects, which may affect the results. (3) Through database retrieval, it is found that at present, this study is more common in China and less in other countries. Also, due to inconsistent outcome indicators, incomplete data, and other factors, the number of included literature is small, and publication bias cannot be avoided. (4) At present, there are few studies on vitamin D adjuvant therapy for NRDS in various countries, so it can not be further meta-analyzed and further verified. (5) Moreover, the various nutritional structures in different countries may lead to the generation of heterogeneity and the instability of results. (6) The selection of cases may also affect the conclusions, as shown by the heterogeneity test, respectively. More nutritional structure-controlled studies were needed to confirm the conclusion of the present meta-analysis.

## Conclusions

This systematic review and meta-analysis study suggests that vitamin D deficiency is very likely to be a high-risk factor of NRDS, but the specific mechanism of vitamin D involved in the occurrence of NRDS disease is still unclear, and further research is needed. Vitamin D deficiency is a global health problem. Although there is currently no unified standard for the prevention and treatment of NRDS with vitamin D supplementation, reasonable vitamin D supplementation during pregnancy and after the birth of newborns is still of great significance to reduce the risk of NRDS, reduce the incidence of complications in children with NRDS and improve the prognosis. In future studies, the therapeutic effect of vitamin D on NRDS and its optimal dose and time should be further explored.

## Data Availability Statement

The raw data supporting the conclusions of this article will be made available by the authors, without undue reservation.

## Author Contributions

YZ lead the study, contributed to team management and coordination, revised the manuscript, polished the language, contributed to the drafting of the articles, and critical revision for important intellectual content. RZ contributed to the data analysis and manuscript formatting. RZ, HZ, XZ, and YL contributed to the literature search, study design, identifying, data acquisition, and recording of the characteristics of studies. RZ and HZ evaluated quality of the cross-sectional studies, reviewed, and rectified the data. RZ, HZ, XZ, YL, and DL contributed to the data interpretation and critical revision to the manuscript. DL embellished the images and downloaded literature. All authors contributed to the article and approved the submitted version.

## Conflict of Interest

The authors declare that the research was conducted in the absence of any commercial or financial relationships that could be construed as a potential conflict of interest.

## Publisher's Note

All claims expressed in this article are solely those of the authors and do not necessarily represent those of their affiliated organizations, or those of the publisher, the editors and the reviewers. Any product that may be evaluated in this article, or claim that may be made by its manufacturer, is not guaranteed or endorsed by the publisher.
